# The distribution of 45S rDNA sites in bird chromosomes suggests multiple evolutionary histories

**DOI:** 10.1590/1678-4685-GMB-2018-0331

**Published:** 2020-03-30

**Authors:** Tiago Marafiga Degrandi, Ricardo José Gunski, Analía del Valle Garnero, Edivaldo Herculano Correa de Oliveira, Rafael Kretschmer, Marcelo Santos de Souza, Suziane Alves Barcellos, Iris Hass

**Affiliations:** 1 Universidade Federal do Paraná (UFPR), Departamento de Genética, Curitiba, PR, Brazil.; 2 Universidade Federal do Pampa (UNIPAMPA), São Gabriel, RS, Brazil.; 3 Universidade Federal do Pará (UFPA), Belém, PA, Brazil.; 4 Instituto Evandro Chagas (IEC), Belém, PA, Brazil.; 5 Universidade Federal do Rio Grande do Sul (UFRGS), Instituto de Biociências, Porto Alegre, RS, Brazil.

**Keywords:** FISH, chromosome, chromosome evolution, cytogenetics, Aves

## Abstract

The distribution of 45S rDNA cluster in avian karyotypes varies in different aspects, such as position, number of bearer chromosomes, and bearers being macro- or microchromosomes. The present study investigated the patterns of variation in the 45S rDNA-bearer chromosomes of birds in order to understand the evolutionary dynamics of the cluster configuration and its contribution to the evolution of bird karyotypes. A total of 73 bird species were analyzed, including both published data and species for which rDNA-FISH was conducted for the first time. In most birds, the 45S rDNA clusters were located in a single pair of microchromosomes. Hence, the location of 45S rDNA in macrochromosomes, observed only in Neognathae species, seems to be a derived state, probably the result of chromosomal fusion between microchromosomes and distinct macrochromosomes. Additionally, the 45S rDNA was observed in multiple microchromosomes in different branches of the bird phylogeny, suggesting recurrence of dispersion processeses, such as duplications and translocations. Overall, this study indicated that the redistribution of the 45S rDNA sites in bird chromosomes followed different evolutionary trajectories with respect to each lineage of the class Aves.

## Introduction

The rDNA genes are extremely important for cell function, given that they encode the rRNA involved in ribosome biogenesis (Hadjiolov, 1985; Shaw and Brown, 2012). In this process, two rDNA clusters are involved: the 45S rDNA composed by 18S, 5.8S, and 28S genes, and internal (ITS1 and ITS2) and external (5’ETS and 3’ETS) transcribed spacers; and the 5S rDNA, composed by a 5S gene separated by an intergenic spacer region (IGS) (Daniels and Delany, 2003; Dyomin *et al.*, 2016). In the eukaryotic genome, multiple copies of these clusters are organized in tandem in the DNA, forming the 5S and 45S rDNA sites in the chromosome (Daniels and Delany, 2003; Dyomin *et al.*, 2016).

Identification of chromosomes that bear 45S rDNA can be performed by the silver nitrate impregnation technique (Ag-NOR) (Howell and Black, 1980). However, this procedure only identifies the chromosomes with 45S rDNA sites in transitional activity, exhibiting intercellular, and interindividual variation (Zurita *et al.*, 1997). In this way, fluorescence *in situ* hybridization (FISH) experiments are more appropriate for this type of study, since they allow the precise identification of the bearing chromosomes when using probes for the genes that make up the rDNA cluster even when they are not active (O’Connor, 2008).

In recent years, FISH has been increasingly used to detect rDNA-bearer chromosomes in a range of vertebrate and invertebrate species (e.g., Roy *et al.*, 2005; Cazaux *et al.*, 2011; Mazzoleni *et al.*, 2018; Sochorová *et al.*, 2018). These studies have shown that 45S and 5S rDNA sites are most frequently found in a single chromosome pair per diploid genome, although considerable variation has been observed, with up to 74 chromosome copies for the 5S rDNA cluster sites and 54 for the 45S (Sochorová *et al.*, 2018). In addition, no significant correlation has been found between the number of 5S and 45S loci, which suggests that their distribution and amplification within the karyotype follow independent evolutionary trajectories (Sochorová *et al.*, 2018).

The location of rDNA sites has been related to hotspots of chromosomal breakage (Cazaux *et al.*, 2011). This fragility is probably originated by the repetitive nature of clusters or their intense gene expression activity (Huang *et al.*, 2008). In the chromosome, these breakages may result in different types of rearrangements, such as translocation, fusions, duplications, and inversions, leading to rapid changes in the chromosomal distribution of the rDNA sites in closely related species (Datson and Murray, 2006; Degrandi *et al.*, 2014).

Birds are a highly diversified biological group with more than 10,000 species. On the other hand, less than 12% of the species have a known karyotype (Kretschmer *et al.*, 2018a). The diploid number ranges from 2 n= 40, as found in *Burhinus oedicnemus*, to 2n = 136-142 in *Corythaixoides concolor* (Christidis, 1990; Nie *et al.*, 2009). However, the karyotype of birds is relatively conserved, and most species have 2n = 80. Generally, their karyotypes are characterized by the presence of macrochromosomes, which are 2.5–6 μm in length, and microchromosomes, which are less than 2.5 μm long (Rodionov, 1996; Kretschmer *et al.*, 2018a). This basic karyotype structure can be seen in the species of both the Paleognathae and Neognathae clades (Kretschmer *et al.*, 2018a).

Studies that have mapped the chromosomal location of 45S rDNA sites have shown considerable divergence among birds (Nishida-Umehara *et al.*, 2007; Nishida *et al.*, 2008, 2013; Nie *et al.*, 2009; Tagliarini *et al.*, 2009; de Oliveira *et al.*, 2013; Kretschmer *et al.*, 2014; Degrandi *et al.*, 2017; de Oliveira *et al.*, 2017). In Paleognathae birds, the 45S rDNA is normally found in a single microchromosome pair (Nishida-Umehara *et al.*, 2007). However, in the Neognathae birds, a significant variation has been observed, including species with 45S rDNA clusters in multiple microchromosomes, in a single macrochromosome pair, or in both (Nishida *et al.*, 2008; de Oliveira *et al.*, 2013; Tagliarini, 2013; Degrandi *et al.*, 2017; de Oliveira *et al.*, 2017). However, the origin of this variation and its possible evolutionary implications are still poorly understood.

Thus, the aim of this study was to investigate this variation in 45S rDNA-bearing chromosomes of birds in order to understand the evolutionary dynamics of the cluster configuration and its contribution to the evolution of the bird karyotype.

## Materials and Methods

### Specimens

In this work, we analyzed the basic karyotype structure and distribution of the 45S rDNA sites in bird karyotypes. The following data were considered in each species: diploid number, number of 45S rDNA-bearing chromosomes, their type (macro- or microchromosome), and position of the clusters on the chromosome arm. First, the data were obtained from the literature, considering only the species in which the 45S rDNA clusters were identified by FISH-rDNA. Ag-NORs data were disregarded due to the intercellular and individual variations or possible false positive results, already reported in the literature.

Additionally, 29 species were selected from the sample bank of the Laboratory of Animal Genetic Diversity at Universidade Federal do Pampa for the first rDNA-FISH screening of each taxon: order Passeriformes/family Thraupidae: *Tachyphonus coronatus*, *Coryphospingus cucullatus*; Icteridae: *Agelaioides badius*, *Molothrus bonariensis*, Tyrannidae: *Pitangus sulphuratus*, *Myiarchus ferox*; Tityridae: *Schiffornis virescens*; Furnariidae: *Dendrocolaptes platyrostris*, *Anumbius annumbi*, *Synallaxis albescens*, *Furnarius rufus*, *Cranioleuca obsoleta*, *Syndactila rufosuperciliata*; Coraciiformes/ Alcedinidae: *Chloroceryle americana*; Piciformes/Ramphastidae: *Ramphastos tucanus*; Accipitriformes/Accipitridae: *Pseudastur albicollis*, *Buteogallus urubitinga*; Pelecaniformes/Ardeidae: *Syrigma sibilatrix*; Charadriiformes/Stercorariidae: *Stercorarius antarcticus*; Caprimulgiformes /Trochilidae: *Amazilia versicolor*, Nyctibiidae: *Nyctibius griseus*, Caprimulgidae: *Hydropsalis torquata*; Cuculiformes/Cuculidae: *Coccyzus melacoryphus*, *Piaya cayana*, *Guira guira*; Columbiformes/Columbidae: *Columbina talpacoti*; Tinamiformes/ Tinamidae: *Nothura maculosa* and *Rhynchotus rufescens* (Table 1).

**Table 1:** Distribution of 45S rDNA clusters in bird karyotypes.

Infraclass/ order	Family	Species	2n	Nº	Type of chromosome	Position	Reference
**Neognathae**							
Passeriformes Oscines	Turdidae	*Turdus rufiventris*	78	6	Micro	NA	Kretschmer *et al.,* 2014
		*Turdus albicollis*	78	4	Micro	NA	Kretschmer *et al.,* 2014
	Thraupidae	*Saltator similis*	80	2	Micro	NA	dos Santos *et al.,* 2015
		*Saltator aurantiirostris*	80	2	Micro	NA	dos Santos *et al.,* 2015
		Tachyphonus coronatus*	80	2	Micro	NA	Present study
		*Coryphospingus cucullatus*	80	2	Micro	NA	Present study
	Icteridae	*Agelaioides badius*	80	4	Micro	NA	Present study
		*Molothrus bonariensis*	80	2	Micro	NA	Present study
	Fringillidae	*Serinus canaria*	80	4	Micro	NA	dos Santos *et al.,* 2017
	Parulidae	*Basileuterus culicivorus*	80	2	Micro	NA	Present study
	Estrildidae	*Taeniopygia guttata*	80	2	Micro	NA	dos Santos *et al.,* 2017
		*Elaenia spectabilis*	80	4	Micro	NA	Kretschmer *et al.,* 2015
Passeriformes Suboscines	Tyrannidae	*Pitangus sulphuratus*	78	2	Micro	NA	Present study
		*Myiarchus ferox*	76	2	Micro	NA	Present study
	Tityridae	*Schiffornis virescens*	82	2	Micro	NA	Present study
	Furnariidae	Dendrocolaptes platyrostris*	82	2	Macro, 1^th^	P	Present study
		*Anumbius annumbi*	82	2	Micro	NA	Present study
		*Synallaxis albescens*	82	2	Micro	NA	Present study
		Furnarius rufus*	82	2	Micro	NA	Present study
		*Cranioleuca obsoleta*	82	2	Micro	NA	Present study
		*Syndactila rufosuperciliata*	82	2	Micro	NA	Present study
Psittaciformes		*Psittacus erithacus*	62-64	8	Micro	NA	Seibold-Torres *et al.,* 2015
Falconiformes	Falconidae	*Falco tinnunculus*	52	4	Micro	NA	Nishida *et al.,* 2008
		*Falco peregrinus*	50	12 or 14	Micro	NA	Nishida *et al.,* 2008
		*Falco columbarius*	40	9	Micro	NA	Nishida *et al.,* 2008
Coraciiformes	Alcedinidae	*Chloroceryle americana*	94	2	Micro	NA	Present study
Piciformes	Picidae	*Colaptes campestres*	84	2	Macro, 13^th^	I	de Oliveira *et al.,* 2017
		*Colaptes melanochloros*	84	2	Macro, 13^th^	I	de Oliveira *et al.,* 2017
		*Melanerpes candidus*	64	2	Micro	NA	de Oliveira *et al.,* 2017
	Ramphastidae	Ramphastos tucanus*	112	2	Micro	NA	Present study
Trogoniformes	Trogonidae	*Trogon s. surrucura*	82	6	Micro	NA	Degrandi *et al.,* 2017
Accipitriformes Eagles	Pandionidae	*Pandion haliaetus*	74	2	Macro, 2^th^	P, q	Nishida *et al.,* 2014
	Accipitridae	*Pseudastur albicollis*	66	2	Macro, 8^th^	P, q	Present study
		Buteogallus urubitinga*	68	2	Macro, 8^th^	P, q	Present study
		*Buteo nitidus*	68	2	Macro, 8^th^	P, q	de Oliveira *et al.,* 2013)
		*Rupornis magnirostris*	68	2	Macro, 8^th^	P, q	de Oliveira *et al.,* 2013
		*Buteogallus meridionalis*	68	2	Macro, 8^th^	P, q	de Oliveira *et al.,* 2013
		*Harpia harpyja*	58	4	Macro, 6^th^ and Micro, 25^th^	S	Tagliarini, 2013
		*Morphnus guianensis*	82	2	Macro, 1^th^	S	Tagliarini, 2013
		*Nisaetus n. orientalis*	66	2	Micro, 29^th^	NA	Nishida *et al.,* 2013
Accipitriformes Vultures	Cathartidae	*Sarcoramphus papa*	80	2	Micro	NA	Tagliarini *et al.,* 2009
		*Cathartes burrovianus*	80	2	Micro	NA	Tagliarini *et al.,* 2009
		*Cathartes aura*	80	2	Micro	NA	Tagliarini *et al.,* 2009
		*Gymnogyps californianus*	80	2	Micro	NA	Raudsepp *et al.,* 2002
Pelecaniformes	Ardeidae	Syrigma sibilatrix*	62	2	Micro	NA	Present study
Charadriiformes	Stercorariidae	*Stercorarius antarcticus*	84	2	Micro	NA	Present study
	Burhinidae	*Burhinus oedicnemus*	42	2	Macro, 13^th^	I	Nie *et al.,* 2009
Caprimulgiformes Hummingbirds	Trochilidae	*Amazilia versicolor*	82	2	Micro	NA	Present study
Caprimulgiformes Nigthjars	Nyctibiidae	*Nyctibius griseus*	*86*	2	Micro	NA	Present study
	Caprimulgidae	*Hydropsalis torquata*	74	2	Micro	NA	Present study
Cuculiformes	Cuculidae	*Coccyzus melacoryphus*	-	2	Micro	NA	Present study
		*Piaya cayana*	88	2	Macro 7^th^	P, p	Present study
		*Guira guira*	76	2	Macro, 6^th^	P, q	Present study
Columbiformes	Columbidae	*Columbina talpacoti*	76	2	Micro	NA	Present study, Kretschemer *et al.,* 2018b
		*Zenaida auriculata*	76	2	Micro	NA	Kretschemer *et al.,* 2018b
		*Geotrygon montana*	86	2	Micro	NA	Kretschemer *et al.,* 2018b
		*Geotrygon violacea*	86	2	Micro	NA	Kretschemer *et al.,* 2018b
		*Leptotila verreauxi*	78	2	Micro	NA	Kretschemer *et al.,* 2018b
		*Patagioenas cayennensis*	76	2	Micro	NA	Kretschemer *et al.,* 2018b
		*Columba livia*	80	2	Micro	NA	Kretschemer *et al.,* 2018b
		*Columbina passerina*	76	2	Micro	NA	Kretschemer *et al.,* 2018b
		*Columbina picui*	76	6	Micro	NA	Kretschemer *et al.,* 2018b
Galliformes	Phasianidae	*Coturnix japonica*	78	6	Micro	NA	McPherson *et al.,* 2014
		*Meleagris gallopavo*	80	2	Micro, 18^th^	NA	McPherson *et al.,* 2014
		*Gallus gallus*	78	2	Micro, 16^th^	NA	Dyomin *et al.,* 2016
**Paleognathae**							
Tinamiformes	Tinamidae	*Nothura maculosa*	78	4	Micro	NA	Present study
		*Eudromia elegans*	80	4	Micro	NA	Nishida-Umehara *et al.,* 2007
		*Rhynchotus rufescens*	78	2	Micro	NA	Present study
Casuariiformes	Dromaiidae	*Dromaius novaehollandiae*	80	2	Micro	NA	Nishida-Umehara *et al.,* 2007
	Casuariidae	*Casuarius casuarius*	92	2	Micro	NA	Nishida-Umehara *et al*., 2007
Struthioniformes	Struthionidae	*Struthio camelus*	80	2	Micro	NA	Nishida-Umehara *et al*., 2007
Rheiformes	Rheidae	*Rhea pennata*	80	2	Micro	NA	Nishida-Umehara *et al.,* 2007
		*Rhea americana*	80	2	Micro	NA	Nishida-Umehara *et al.,* 2007

**Figure 1:**
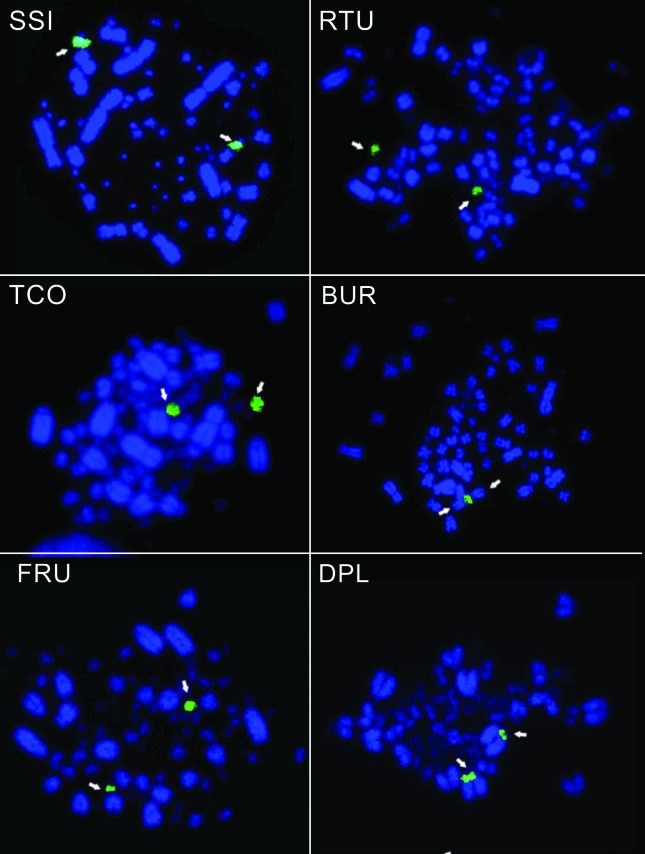
Examples of the metaphases analyzed in the present study using the 18S rDNA probe (green) to identify the chromosomes (blue) carrying 45S rDNA sites (arrows). The acronym shown in the upper right corner of each metaphase indicates the species: *Syrigma sibilatrix* (SSI), *Ramphastos tucanus* (RTU), *Tachyphonus coronatus* (TCO), *Buteogallus urubitinga* (BUR), *Furnarius rufus* (FRU), and *Dendrocolaptes platyrostris* (DPL).

2n = diploid number; Nº = Number of 45S rDNA-bearing chromosomes; Type of chromosome: Macro = Macrochromosome; Micro = Microchromosome; Nomenclature for the position in the chromosome: I= Interstitial; S = subtelomeric; P = Pericentromeric; Arm location: Short arm = p; Long arm = q; NA = Not applicable; *Shown in the fig. 1; Species names are in accordance with Gill and Donsker (2018).

### Chromosome preparation

Mitotic chromosomes were obtained following standard protocols, including direct preparation from bone marrow, fibroblast culture, and lymphocyte culture (Moorhead *et al.*, 1960; Sasaki *et al.*, 1968; Garnero and Gunski, 2000).

### FISH for 18S rDNA

FISH using probes specific for the 18S rDNA gene identified the 45S rDNA-bearing chromosomes. Primers were developed from sequences obtained from the fish *Hoplias malabaricus* (Cioffi *et al.*, 2009). This generated a fragment of approximately 1,400 base pairs, which was labeled by polymerase chain reaction (PCR), using the primers 18SF (5’CCGAGGACCTCACTAAACCA 3’) and 18SR (5’CCGCTTTGGTGACTCTTGAT-3’), with fluorescein dUTP in the PCR mix.

The PCRs were run in a final volume of 25 μL containing 2 ng of genomic DNA from *H*. *malabaricus*, 0.2 μM of each primer (18SF and 18SR), 0.2 mM of dNTP, 10X buffer (1x), 50 mM of MgCl_2_ (2 μM), 1 mM of Fluorescein-12-dUTP solution, 1 U/μL of *Taq* polymerase, and sterile H_2_O to complete to final volume. The thermal cycling parameters were 94 ºC for 60 s, 30 cycles of 94 ºC for 60 s, 60 ºC for 60 s, 72 ºC for 90 s, followed by an elongation step of 5 min at 72 ºC (Cioffi *et al.*, 2009).

For the FISH procedures, slides with metaphases were treated with RNase A (10 μg/mL) for 20 min and then denatured in 70% formamide at 70°C for 80 s. Subsequently, 300 ng of the 18S probe were added to each slide, which was then sealed with a cover slip and incubated overnight at 37 °C (Daniels and Delany, 2003). The slides were then washed in 50% formamide at 42 °C for 1 min (x2), 2xSSC at 40 °C for 2.5 min (x2), and once in 4xSSC Tween (1X) at room temperature. The chromosomes were counterstained with DAPI. Hybridization results were analyzed using a Zeiss Axioplan2 fluorescence microscope.

### Chromosomal analyses

The diploid number of each specimen was determined from the analysis of approximately 30 mitotic cells stained with Giemsa observed under an optical microscope. Variation in the number of rDNA clusters was evaluated based on the number of chromosomes that presented a fluorescent signal. The rDNA cluster-bearing chromosomes were classified as either macrochromosomes or microchromosomes, according to their length (Rodionov, 1996). The position of the 45S rDNA cluster was classified as: (i) pericentromeric (adjacent to the centromere), (ii) subtelomeric (adjacent to the telomere), and (iii) interstitial (between the centromere and the telomere) (Cazaux *et al.*, 2011). Ideograms were created using these characteristics to represent the rDNA-bearing chromosomes in each species.

### Phylogenetic comparison

The species were compared using the phylogenetic relationships proposed by Jarvis *et al.* (2014) and Prum *et al.* (2015). In this step, the chromosomal locations of the 45S rDNA clusters were plotted in a modified phylogenetic tree of Jarvis *et al.* (2014). In this tree, we used the Mesquite software to exclude groups of birds for which rDNA location data were not available. We considered the presence of 45S rDNA in a single pair of microchromosomes as an ancestral condition for birds, according to the hypothesis of Nishida-Umehara *et al.* (2007). Based on this hypothesis, we analyzed the evolutionary relationships and the probable chromosomal rearrangements that would explain the variations observed in chromosomes carrying 45S rDNA.

## Results

The number of chromosomes (2n), number of 45S rDNA sites, and the characteristics of these bearing chromosomes from 29 selected species for rDNA-FISH screening in this work are shown in Table 1 (see species identified as ‘present study’ in Table 1). The rDNA-FISH results of some selected species are shown in Figure 1.

Overall, the analysis of the chromosomal distribution of the 45S rDNA included 73 bird species, representing 17 orders of the class Aves (Table 1). Eight of these species were Paleognaths, representing four orders, the Casuariiformes, Rheiformes, Struthioniformes, and Tinamiformes. The other 65 species were Neognaths, belonging to 13 orders, the Accipitriformes, Caprimulgiformes, Charadriiformes, Columbiformes, Coraciiformes, Cuculiformes, Falconiformes, Galliformes, Passeriformes, Pelecaniformes, Piciformes, Psittaciformes, and Trogoniformes.

### Variation in the diploid number in birds

**Figure 2:**
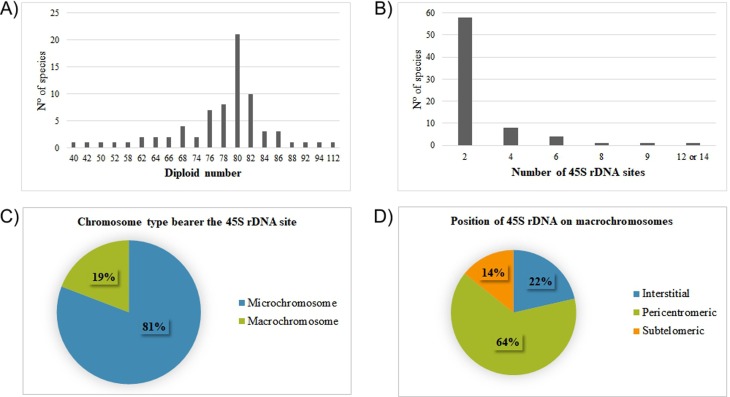
Chromosomal location of the 45S rDNA sites in all 73 bird species analyzed in the present study. (A) variation in the diploid number; (B) variation in the number of 45S rDNA bearer chromosomes; (C) the proportion of the species with 45S rDNA located in macrochromosomes or microchromosome; (D) location of the 45S rDNA cluster in the chromosome arm.

Considering only the bird species for which the location of 45S rDNA sites is available (73), diploid numbers ranged from 2n = 40 to 2n = 112 (Table 1). Despite this ample variation, most (38) of the species had diploid numbers between 78 and 82, and 21 were 2n = 80 (Figure 2A). While the Paleognathae species were relatively conserved, with most species having around 80 chromosomes, higher variability in 2n was observed in Neognathae (Table 1).

### Number of 45S rDNA sites

The analysis of the number of 45S rDNA-bearing chromosomes highlighted that most (58) species had a cluster in a single chromosome pair (Figure 2B). In the Paleognathae, *Nothura maculosa* and *Eudromia elegans* were exceptions, with two rDNA-bearing chromosome pairs. In the Neognathae, the 45S rDNA clusters were found in a single chromosome pair and in up to six or seven pairs (Table 1).

### Types of rDNA-bearing chromosomes

In the bimodal analysis of macrochromosomes *vs.* microchromosomes, the 45S rDNA sites of most (59) species were observed on microchromosomes (Figure 2C). In the Paleognathae, the rDNA was located exclusively on microchromosomes. The Neognathae presented different configurations, by contrast, with some species having the cluster in the microchromosomes, others in the macrochromosomes, and some in both types of chromosome, as observed in the Accipitriformes, *Harpia harpija* (Table 1).

The location of the rDNA in macrochromosomes was observed in 14 Neognathae species (Table 1), representing a number of different orders: *Pandion haliaetus*, *Pseudastur albicollis*, *Buteogallus urubitinga*, *Buteo nitidus*, *Rupornis magnirostris*, *B. meridionalis*, *H. harpyja* and *Morphnus guianensis* (Accipitriformes), *Burhinus oedicnemus* (Charadriiformes), *Piaya cayana* and *Guira guira* (Cuculiformes), *Dendrocolaptes platyrostris* (Passeriformes), *Colaptes campestres*, and *Colaptes melanochloros* (Piciformes). In some cases, it was possible to identify homologies between the macrochromosomes and those of *Gallus gallus* (Table 2).

**Table 2:** Associations of 45S rDNA sites with macrochromosomes and their respective homologies with *Gallus gallus* (GGA) chromosomes.

Order	Species	45S rDNA chromosome location	Homologous GGA segment *	Reference
Accipitriformes	*Pandion haliaetus*	2^th^	GGA1	Nishida *et al.,* 2014
	*Harpia harpyja*	6^th^ and 25^th^	GGA1	Tagliarini, 2013
	*Morphnus guianensis*	1^th^	GGA3	Tagliarini, 2013
	*Pseudastur albicollis*	8^th^	GGA7	de Oliveira *et al*., 2010
	*Buteo nitidus*	8^th^	GGA7	de Oliveira *et al.,* 2013
	*Rupornis magnirostris*	8^th^	GGA7	de Oliveira *et al.,* 2013
	*Buteogallus meridionalis*	8^th^	GGA7	de Oliveira *et al.,* 2013
Charadriiformes	*Burhinus oedicnemus*	13^th^	2 Micro	Nie *et al.,* 2009
Cuculiformes	*Piaya cayana*	7^th^	GGA2	Unpublished data
	*Guira guira*	6^th^	GGA2	Unpublished data

* Homologies established by chromosome painting; Micro: Microchromosome.

### Position of the 45S rDNA site in the chromosomes

As microchromosomes have a limited resolution, the species with rDNA sites in these tiny elements were excluded from the analysis of the rDNA topology in the chromosomes in order to avoid biases in data interpretation. Therefore, the position of the rDNA cluster was analyzed only in the 14 species in which the 45S rDNA is located in macrochromosomes.

The 45S rDNA was observed in a pericentromeric position in most (64%) cases, that is, in *P*. *haliaetus*, *P*. *albicollis*, *B*. *urubitinga*, *B*. *nitidus*, *R*. *magnirostris*, *B*. *meridionalis* (Accipitriformes), *G*. *guira*, *P. cayana* (Cuculiformes), and *D*. *platirostris* (Passeriformes). The interstitial position was the second most frequent, being observed in 22% of the species, *B*. *oedicnemus* (Charadriiformes), *C*. *campestres*, and *C*. *melanochloros* (Piciformes). Finally, a subtelomeric position was recorded in two (14%) species, *M*. *guianensis* and *H*. *harpyja* (Accipitriformes) (Figure 2D, Table 1).

### Phylogenetic comparisons

**Figure 3:**
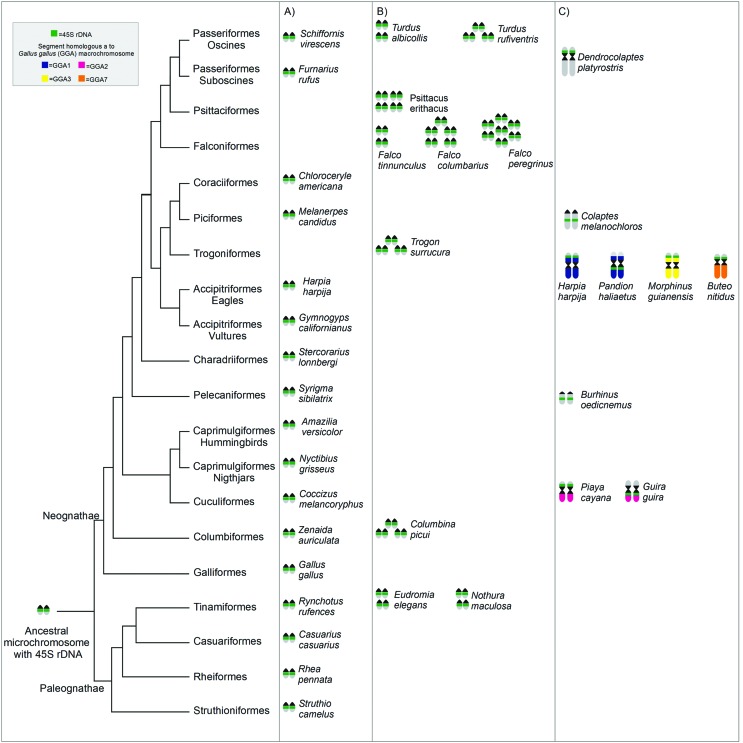
Phylogenetic relationships among the birds modified from Jarvis *et al.*, 2014. The data of chromosomal location of the 45S rDNA from species analyzed in the present study were plotted in the tree. (A) Species with rDNA located only in a microchromosome pair; (B) species with rDNA in multiples microchromosomes; (C) species in which the rDNA is located in macrochromosomes. The complete data are shown in Table 1.

For the phylogenetic comparisons, the presence of the 45S rDNA cluster in a single pair of microchromosomes was considered to be the ancestral condition, based on the hypothesis of Nishida-Umehara *et al.* (2007). This analysis revealed that the variation in the number of 45S rDNA-bearer chromosomes was independent of the phylogenetic relationships among the species (Figure 3). The presence of rDNA in macrochromosomes was observed in species belonging to different orders from infra class Neognathae (Figure 3).

## Discussion

Here we present for the first time a broad analysis of the distribution of 45S rDNA in avian karyotype. Although an impressive variation was observed in the chromosomes carrying the 45S rDNA cluster, we recorded that in most species it is located in a single pair of microchromosomes. Interestingly, most of these species have a karyotype with 2n = 80 chromosomes (Figure 2A).

A study with rodents indicated that there is no relationship between the 2n and the number of 45S rDNA cluster bearing chromosomes (Cazaux *et al.*, 2011). Nevertheless, birds with 2n = 80 chromosomes that carry only a single pair of 45S rDNA microchromosomes seem to reflect the karyotype conservation status of these species in relation to the ancestral karyotype of birds (PAK), as proposed by Griffin *et al.* (2007). This karyotype uniformity of birds has also been observed in species from Paleognathae and Neognathae using the GGA whole chromosome paint (Kretschmer *et al.*, 2018a).

The presence of a single pair of microchromosomes with 45S rDNA conserved among the species of Paleognaths (*Dromaius novaehollandiae*, *Casuarius casuarius*, *Struthio camelus*, *Rhea pennata*, and *Rhea americana*) suggests that this would be an ancestral condition of rDNA (Nishida-Umehara *et al.*, 2007). Using the phylogenetic relationships proposed by Jarvis *et al.* (2014) and Prum *et al.* (2015), we compared these data and identified that several species of the Neognaths infraclass preserve the 45S rDNA in a pair of microchromosome (Figure 3), a fact that reinforces the hypothesis of PAK ancestral condition (Griffin *et al.*, 2007).

The 45S rDNA-bearer chromosome is related to the presence or absence of the process of karyotypic diversification. For example, Accipitriformes, where species of the Cathartidae family have karyotypes with 80 chromosomes, the 45S rDNA was located in only a single pair of microchromosomes (Raudsepp *et al.*, 2002; Tagliarini *et al.*, 2009). In contrast, the Accipitridae family shows a diploid number quite derived (2n = 58-82), and chromosome painting evidenced an extensive karyotypic reorganization, originated by breaks and fusions of macrochromosomes (GGA) and microchromosomes. In this group, it was observed that 45S rDNA is associated with different macrochromosomes (Table 2) (de Oliveira *et al.*, 2013, Tagliarini, 2013; Nishida *et al.*, 2014).

### 45S rDNA in multiple microchromosomes

Multiple microchromosomes carrying 45S rDNA can be found in some species of the orders Tinamiformes, Columbiformes, Trogoniformes, and Falconiformes, and notably, even phylogenetically related species may differ in the number of rDNA bearing chromosomes. For instance, Paleognath birds from the order Tinamiformes show variation in the number of clusters. In *R. rufescens* a single microchromosome pair containing the 45S rDNA was observed, whereas in *N. maculosa* and *E. elegans*, the 45S rDNA is located in two pairs of microchromosomes (Figure 3). Similarly, such numerical variation is also seen in species of the same genus, as in the genus *Falco* (Falconiformes), where *F. tinnunculus* has 45S rDNA in four microchromosome pairs, *F. columbarius* in five pairs, and *F. peregrinus* shows this cluster in six or seven pairs (Nishida *et al.*, 2008) (Figure 3). Considering the phylogenetic relationships between these orders, the most plausible explanation for the origin of these variation are recurrent processes of 45S rDNA cluster duplications or translocations, resulting in the numerical variation observed in these species.

### 45S rDNA distribution in macrochromosomes

The 45S rDNA location in macrochromosomes can be considered a derived characteristic in birds (Kretschmer *et al.*, 2018a). The available data on chromosomal homologies with *G. gallus* (GGA) (Table 2), demonstrated that the rDNA sites are clearly associated with distinct macrochromosomes. This scenario might have been originated by multiple independent events of chromosomal fusion, which are supported by several different types of evidence.

In Accipitriformes, for example, multiple associations were recorded, including GGA1, GGA3, and GGA7. In *B. nitidus*, *R. magnirostris*, and *B. meridionallis*, an association with the homologous GGA7 segment was found, although the short arm of the chromosome pair containing the rDNA of these species was not hybridized by any of the GGA probes used (de Oliveira *et al.*, 2013). This unhybridized region probably corresponds to the homologous of the ancestral microchromosome containing the rDNA, reinforcing the fusion hypothesis. Similarly, in *P. haliaetus*, the rDNA located on the q-arm of chromosome 2 was associated with the homologous GGA1 segment (Nishida *et al.*, 2014). In this species, the short arm did not hybridize by any GGA probe. However, *P. haliaetus* showed rDNA in the long arm, suggesting that a pericentric inversion should have occurred after fusion with the 45S rDNA microchromosome, shifting the cluster position to the long arm.

### 45S rDNA related to intrachromosomal rearrangements

Intrachromosomal rearrangements have been reported in bird karyotypes, and our data revealed that two cases involved the 45S rDNA-bearer chromosome (Degrandi *et al.*, 2017). For example, in Cuculiformes, *Piaya caiana* and *Guira guira* showed the association of 45S rDNA with a segment homologue to chromosome GGA2 (Table 2). In *P. caiana*, the cluster was in the pericentromeric region of the short arm of the submetacentric chromosome pair 7, whereas in *G. guira* the cluster was in the long arm pericentromeric region of the metacentric chromosome 6 (Figure 3). In Accipitriformes *Harpia harpyja* and *Pandion haliaetus*, the association was with a segment homologue to chromosome GGA1 (Table 2). However, in *H. harpyja*, the rDNA cluster was seen in the subtelomeric region of macrochromosome 6, and in *P. haliaetus*, the cluster occupied the pericentromeric region of the long arm on chromosome 2 (Figure 3) (Tagliarini, 2013; Nishida *et al.*, 2014). The translocation or a pericentric inversion may explain this position variation of the internal 45S rDNA cluster in the bearer chromosome, which corroborates the hypothesis that the 45S rDNA cluster is related to chromosomal breakpoints, according to Cazaux *et al.* (2011).

## Conclusion

In birds, the 45S rDNA site is located predominantly in a single pair of microchromosomes, although a number of deviations from this basic pattern exist, with some species having rDNA located in more than one microchromosome pair or in macrochromosomes, or in both types of chromosome. The present study also demonstrated that the redistribution of rDNA sites within the chromosome complement has resulted from chromosomal rearrangements, which have resulted from the distinct evolutionary histories of each group of the class Aves.

## References

[B1] Cazaux B, Catalan J, Veyrunes F, Douzery EJ, Britton-Davidian J (2011). Are ribosomal DNA clusters rearrangement hotspots? a case study
in the genus *Mus* (Rodentia, Muridae). BMC Evol Biol.

[B2] Christidis L (1990). Animal cytogenetics 4: Chordata 3 B: Aves.

[B3] Cioffi MB, Martins C, Centofante L, Jacobina U, Bertollo LAC (2009). Chromosomal variability among allopatric populations of
Erythrinidae fish *Hoplias malabaricus*: Mapping of three
classes of repetitive DNAs. Cytogenet Genome Res.

[B4] Daniels LM, Delany ME (2003). Molecular and cytogenetic organization of the 5S ribosomal DNA
array in chicken (*Gallus gallus*). Chromosome Res.

[B5] Datson PM, Murray BG (2006). Ribosomal DNA locus evolution in Nemesia: transposition rather
than structural rearrangement as the key mechanism?. Chromosome Res.

[B6] Degrandi TM, Pita S, Panzera Y, de Oliveira EHC, Marques JRF, Figueiró MR, Marques LC, VInadé L, Gunski RJ, Del Valle Garnero A (2014). Karyotypic evolution of ribosomal sites in buffalo subspecies and
their crossbreed. Genet Mol Biol.

[B7] Degrandi TM, Garnero ADV, O’Brien PCM, Ferguson-Smith MA, Kretschmer R, de Oliveira EHC, Gunski RJ (2017). Chromosome painting in *Trogon s. surrucura*
(Aves, Trogoniformes) reveals a karyotype derived by chromosomal fissions,
fusions, and inversions. Cytogenet Genome Res.

[B8] de Oliveira EHC, Tagliarini MM, Rissino JD, Pieczarka JC, Nagamachi CY, O’Brien PCM, Ferguson-Smith MA (2010). Reciprocal chromosome painting between white hawk
(*Leucopternis albicollis*) and chicken reveals extensive
fusions and fissions during karyotype evolution of Accipitridae (Aves,
Falconiformes). Chromosome Res.

[B9] de Oliveira EHC, Tagliarini MM, dos Santos MS, O’Brien PCM, Ferguson-Smith MA (2013). Chromosome painting in three species of Buteoninae: A cytogenetic
signature reinforces the monophyly of south American species. PLoS One.

[B10] de Oliveira TD, Kretschmer R, Bertocchi NA, Degrandi TM, de Oliveira EHC, Cioffi MDB, Garnero AD, Gunski RJ (2017). Genomic organization of repetitive DNA in woodpeckers (Aves,
Piciformes): implications for karyotype and ZW sex chromosome
differentiation. PLoS One.

[B11] dos Santos MS, Kretschmer R, Silva FA, Ledesma MA, O’Brien PCM, Ferguson-Smith MA, Del Valle Garnero A, de Oliveira EH, Gunski RJ (2015). Intrachromosomal rearrangements in two representatives of the
genus *Saltator* (Thraupidae, Passeriformes) and the
occurrence of heteromorphic Z chromosomes. Genetica.

[B12] dos Santos MDS, Kretschmer R, Vilches CF, Bakker A, Gahr M, O’Brien PCM, Ferguson Smith MA, de Oliveira EHC (2017). Comparative cytogenetics between two important songbird, models:
the zebra finch and the canary. PLoS One.

[B13] Dyomin AG, Koshel EI, Kiselev AM, Saifitdinova AF, Galkina SA, Fukagawa T, Kostareva AA, Gagiskaya ER (2016). Chicken rRNA gene cluster structure. PLoS One.

[B14] Garnero ADV, Gunski RJ (2000). Comparative analysis of the karyotypes of *Nothura
maculosa* and *Rynchotus rufescens* (Aves:
Tinamidae). A case of chromosomal polymorphism. Nucleus.

[B15] Griffin DK, Robertson LB, Tempest HG, Skinner BM (2007). The evolution of the avian genome as revealed by comparative
molecular cytogenetics. Cytogenet Genome Res.

[B16] Hadjiolov AA (1985). The Nucleolus and Ribosome Biogenesis.

[B17] Howell WM, Black DA (1980). Controlled silver staining of nucleolus organizer regions with a
protective colloidal developer: a 1-step method. Experientia.

[B18] Huang J, Ma L, Yang F, Fei SZ, Li L (2008). 45S rDNA regions are chromosome fragile sites expressed as gaps
in vitro on metaphase chromosomes of root-tip meristematic cells in
*Lolium spp*. PLoS One.

[B19] Jarvis ED, Mirarab S, Aberer AJ, Li B, Houde P, Li C, Ho SYW, Faircloth BC, Nabholz B, Howard JT (2014). Whole-genome analyses resolve early branches in the tree of life
of modern birds. Science.

[B20] Kretschmer R, Gunski RJ, Garnero ADV, Furo IDO, O’Brien PCM, Ferguson-Smith MA, de Oliveira EH (2014). Molecular cytogenetic characterization of multiple
intrachromosomal rearrangements in two representatives of the genus
*Turdus* (Turdidae, Passeriformes). PLoS One.

[B21] Kretschmer R, de Oliveira EHC, dos Santos MS, Furo IDO, O’Brien PCM, Ferguson-Smith MA, Garnero del Valle, Gunski RJ (2015). Chromosome mapping of the large Elaenia (*Elaenia
spectabilis*): evidence for a cytogenetic signature for
passeriform birds?. Biol J Linn Soc.

[B22] Kretschmer R, Ferguson-Smith MA, de Oliveira EHC (2018). Karyotype evolution in birds: From conventional staining to
chromosome painting. Genes.

[B23] Kretschmer R, de Oliveira TD, Furo IO, Silva FAO, Gunski RJ, Garnero AV, Cioffo MB, de Oliveira EHC, de Freitas TRO (2018). Repetitive DNAs and shrink genomes: a chromosomal analysis in
nine Columbidae species (Aves, Columbiformes). Genet Mol Biol.

[B24] Mazzoleni S, Rovatsos M, Schillaci O, Dumas F (2018). Evolutionary insight on localization of 18S, 28S rDNA genes on
homologous chromosomes in Primates genomes. Comp Cytogenet.

[B25] McPherson MC, Robinson CM, Gehlen LP, Delany ME (2014). Comparative cytogenomics of Poultry: Mapping of single gene and
repeat loci in the Japanese quail (*Coturnix
japonica*). Chromosome Res.

[B26] Moorhead OS, Nowell PC, Mellinan WJ, Battips DM, Ungerford DA (1960). Chromossome preparations of leukocytes cultured from human
peripheral blood. Exp Cell Res.

[B27] Nie W, O’Brien PCM, Fu BL, Beiyuan NG, Vitaly V, Carter NP, Ferguson-Smith MA, Yang F (2009). Avian comparative genomics: reciprocal chromosome painting
between domestic chicken (*Gallus gallus*) and the stone
curlew (*Burhinus oedicnemus* Charadriiformes) - an atypical
species with low diploid number. Chromosome Res.

[B28] Nishida C, Ishijima J, Kosaka A, Tanabe H, Habermann FA, Griffin DK, Matsuda Y (2008). Characterization of chromosome structures of Falconinae
(Falconidae, Falconiformes, Aves) by chromosome painting and delineation of
chromosome rearrangements during their differentiation. Chromosome Res.

[B29] Nishida C, Ishijima J, Ishishita S, Yamada K, Griffin DK, Yamazaki T, Matsuda Y (2013). Karyotype reorganization with conserved genomic
compartmentalization in dot-shaped microchromosomes in the Japanese mountain
Hawk-eagle (*Nisaetus nipalensis orientalis*
Accipitridae). Cytogenet Genome Res.

[B30] Nishida C, Ishishita S, Yamada K, Griffin DK, Matsuda Y (2014). Dynamic chromosome reorganization in the Osprey (*Pandion
haliaetus* Pandionidae, Falconiformes): Relationship between
chromosome size and the chromosomal distribution of centromeric repetitive
DNA sequences. Cytogenet Genome Res.

[B31] Nishida-Umehara C, Tsuda Y, Ishijima J, Ando J, Fujiwara A, Matsuda Y, Griffin K (2007). The molecular basis of chromosome orthologies and sex chromosomal
differentiation in Palaeognathous birds. Chromosome Res.

[B32] O’Connor C (2008). Fluorescence *in situ* hybridization
(FISH). Nat Educ.

[B33] Prum RO, Berv JS, Dornburg A, Field DJ, Townsend JP, Lemmon EM, Lemmon AR (2015). A comprehensive phylogeny of birds (Aves) using targeted
next-generation DNA sequencing. Nature.

[B34] Raudsepp T, Houck ML, O’Brien PMC, Ferguson-Smith MA, Ryder OA, Chowdhary BP (2002). Cytogenetic analysis of California Condor (*Gymnogyps
californianus*) chromosomes: comparison with chicken
(*Gallus gallus*) macrochromosomes. Cytogenet Genome Res.

[B35] Rodionov AV (1996). Micro vs. macro: A review of structure and functions of avian micro- and
macrochromosomes. Russ J Genet.

[B36] Roy V, Monti-Dedieu L, Chaminade N, Siljak-Yakovlev S, Aulard S, Lemeunier F, Montchamp-Moreau C (2005). Evolution of the chromosomal location of rDNA genes in two
*Drosophila* species subgroups:
*ananassae* and *melanogaster*. Heredity.

[B37] Sasaki M, Ikeuchi T, Maino S (1968). A feather pulp culture for avian chromosomes with notes on the
chromosomes of the peafowl and the ostrich. Experientia.

[B38] Seibold-Torres C, Owens E, Chowdhary R, Ferguson-Smith MA, Tizard I, Raudsepp T (2015). Comparative cytogenetics of the Congo African Grey Parrot
(*Psittacus erithacus*). Cytogenet Genome Res.

[B39] Shaw P, Brown J (2012). Nucleoli: Composition, function, and dynamics. Plant Physiol.

[B40] Sochorová J, Garcia S, Gálvez F, Symonová R, Kovarík A (2018). Evolutionary trends in animal ribosomal DNA loci. introduction to a new online database. Chromosoma.

[B41] Tagliarini MM, Pieczarka JC, Nagamachi CY, Rissino J, de Oliveira EHC (2009). Chromosomal analysis in Cathartidae: Distribution of
heterochromatic blocks and rDNA, and phylogenetic
considerations. Genetica.

[B42] Zurita F, Sánchez A, Burgos M, Jiménez R, de la Guardia RD (1997). Interchromosomal, intercellular and interindividual variability
of NORs studied with silver staining and in situ
hybridization. Heredity.

